# Characterization of the hepatitis C virus epidemic in Pakistan

**DOI:** 10.1186/s12879-019-4403-7

**Published:** 2019-09-14

**Authors:** Sarwat Mahmud, Zaina Al Kanaani, Laith J. Abu-Raddad

**Affiliations:** 1 Infectious Disease Epidemiology Group, Weill Cornell Medicine-Qatar, Qatar Foundation - Education City, Cornell University, P.O. Box 24144, Doha, Qatar; 2 Department of Healthcare Policy & Research, Weill Cornell Medicine, Cornell University, New York, USA; 3 College of Health and Life Sciences, Hamad bin Khalifa University, Doha, Qatar

**Keywords:** HCV, Hepatitis C, Pakistan, Province, Genotype, Prevalence, Epidemic

## Abstract

**Background:**

With one in every 20 Pakistanis already infected, Pakistan has the second largest number of hepatitis C virus (HCV) infections globally. The aim of this study was to present a quantitative and analytical characterization of the HCV epidemic in Pakistan.

**Methods:**

A standardized database of HCV antibody incidence and prevalence and HCV genotypes in all subpopulations was systematically assembled. Random-effects meta-analyses and random-effects meta-regressions were performed. Shannon Diversity Index was calculated to determine genotype diversity.

**Results:**

The database included two incidence, 309 prevalence, and 48 genotype measures. Pooled mean HCV prevalence ranged between 7.0% (95% confidence interval (CI): 5.8–8.3%) in Sindh and 0.9% (95% CI: 0.1–2.4%) in Federally Administered Tribal Areas (F.A.T.A). Estimated number of chronically-infected persons ranged between 4.2 million in Punjab and 0.03 million in F.A.T.A. HCV prevalence was stable over time [adjusted odds ratio (AOR) of 1.0 (95% CI: 1.0–1.0)]. Population classification was the strongest predictor of HCV prevalence, explaining 51.8% of prevalence variation. Relative to the general population, HCV prevalence was higher in people who inject drugs [AOR of 23.8 (95% CI: 13.0–43.6)], populations with liver-related conditions [AOR of 22.3 (95% CI: 15.7–31.6)], and high-risk clinical populations [AOR of 7.8 (95% CI: 4.8–12.7)]. Low genotype diversity was observed (Shannon diversity index of 0.67 out of 1.95; 34.5%). There were only minor differences in genotype diversity by province, with genotype 3 being most common in all provinces.

**Conclusion:**

Pakistan’s HCV epidemic shows homogeneity across the provinces, and over time. HCV prevalence is strikingly persistent at high level, with no evidence for a decline over the last three decades. Scale up of HCV treatment and prevention is urgently needed.

**Electronic supplementary material:**

The online version of this article (10.1186/s12879-019-4403-7) contains supplementary material, which is available to authorized users.

## Background

The blood-borne pathogen, hepatitis C virus (HCV), chronically infects approximately 62–79 million persons worldwide [1, 2]. HCV infection is one of the causes of several morbidities including fibrosis, cirrhosis, and liver cancer, placing a strain on healthcare systems [2–5]. The recently available and highly efficacious direct-acting antivirals (DAA) can treat the infection and reduce its disease burden [6]. As such, global targets for elimination of HCV infection as a global health concern by 2030 have been set by the World Health Organization (WHO) [7, 8].

With one in every 20 Pakistanis being infected [9, 10], Pakistan has the second largest number of HCV infections globally [11]. Ongoing transmission appears common, with most infections apparently resulting from healthcare-related exposures, such as poor sterilization of medical equipment and therapeutic injections, among others [9]. Achieving the WHO targets for elimination entails an in depth and analytical characterization of HCV epidemiology in Pakistan, both at the national and regional levels, to develop cost-effective and targeted prevention and treatment interventions. Nonetheless, only one nationally-representative population-based survey has been conducted in Pakistan, and over a decade ago [12].

In this study, utilizing and updating an extensive database of HCV measures that was assembled recently through a systematic review of HCV antibody prevalence (the prevalence of HCV antibody-positive serum, hereinafter referred to as HCV prevalence) in Pakistan [9], we provide a comprehensive analytical and quantitative characterization of diverse aspects of the epidemic in this country. Specifically, we 1) assess the geographical distribution of infection across Pakistan’s provinces, 2) estimate the number of HCV antibody positive persons and the number of HCV chronically-infected persons across Pakistan’s provinces, 3) identify the predictors of HCV prevalence and sources of between-study heterogeneity, 4) determine (importantly) the trend in the HCV epidemic over the last three decades, and 5) calculate the HCV genotype distribution and its diversity across Pakistan’s provinces. Accordingly, we provide for the first time such detailed and extensive analytics of this epidemic, the second largest globally.

This work was conducted as part of the Middle East and North Africa (MENA) HCV Epidemiology Synthesis Project [9, 10, 13–28], an ongoing effort to characterize HCV epidemiology and inform key public health research, policy, and programming priorities in the region.

## Methods

### Systematic review of HCV incidence and prevalence

We updated and expanded an HCV incidence and prevalence systematic review that was published previously [9]. In addition to this update to include two additional years of data, we extracted all HCV ribonucleic acid (RNA) measures in Pakistan. We further conducted a systematic review of HCV genotypes. The systematic review methodology in both of these systematic reviews followed that used in the previous systematic review [9], and the other systematic reviews of the HCV Synthesis Project [9, 13–20]. Full details of the methodology are available in these previous publications [9, 13–20].

In brevity, all records on Pakistan including HCV incidence or prevalence measures up to 19th of March, 2018, were systematically reviewed and included in the present study, guided by the Cochrane Collaboration Handbook [29]. Preferred Reporting Items for Systematic Reviews and Meta-analyses (PRISMA) guidelines were used in reporting the results (Additional file 1: Table S1) [30]. Systematic literature searches were performed on PubMed and Embase, using broad search criteria and no language restrictions (Additional file 1: Figure S1).

Duplicates were excluded and remaining unique citations titles and abstracts were screened for relevance. Full-texts of reports determined to be either relevant or potentially relevant underwent additional screening. The references of all reviews and included reports were also screened for additional sources of data that may have been overlooked. Any document listing primary data on HCV incidence or prevalence antibody measures was included. All articles, regardless of reporting HCV incidence or prevalence, were included in an additional independent systematic screening for HCV genotype information.

**Fig. 1:**
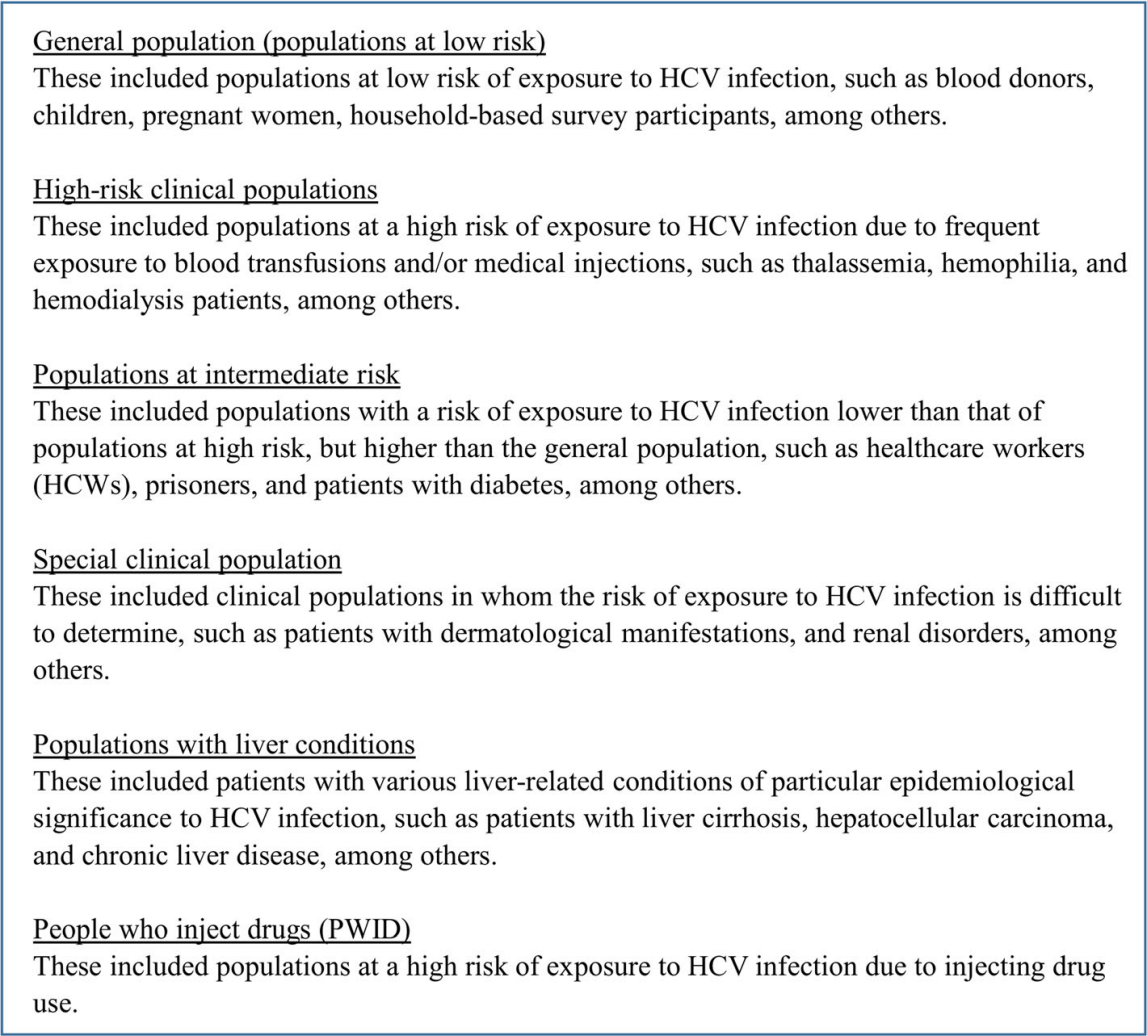
Population classification into categories based on risk of exposure to hepatitis C virus (HCV) infection

Extracted data were synthesized into six population categories defined by exposure risk to the infection, as informed by HCV epidemiology literature [11, 31, 32], and the earlier studies of the HCV Synthesis Project [9, 13–20]. Risk categories and classification of identified populations is presented in Fig. 1.

Pakistan has recently passed an amendment to merge Federally Administered Tribal Areas (F.A.T.A) and Khyber Pakhtunkhwa into one province, a process that will take a year to be completed [33]. For the present study, F.A.T.A and Khyber Pakhtunkhwa were reported and analyzed as two separate provinces.

### Pooled mean HCV prevalence in the general population

The pooled mean HCV prevalence in the general population across Pakistan’s provinces was estimated by performing meta-analyses whenever ≥3 prevalence measures were available. Studies with ≥25 participants qualified to be included in meta-analysis. Stratified prevalence measures replaced the prevalence for the full sample provided the subsample size (for each stratum) was ≥25 participants. Eventually, one stratification was used for each study based on a priori sequential order, prioritizing population, province, sex, year, and age.

HCV prevalence variance was stabilized using the Freeman-Tukey type arcsine square-root transformation [34]. HCV prevalence (with inverse variance weighting) was pooled using a DerSimonian-Laird random-effects model [35]. Heterogeneity was characterized using several statistical measures [35, 36].

Analyses were performed using R version 3.4.3 [37], with all spatial mapping being performed in Tableau 10.1 [38].

### Number of HCV infected persons

The number of HCV antibody positive persons in each province was assessed through the product of the province-specific pooled mean HCV prevalence and the population size in each province. This was then multiplied by the pooled mean fraction of HCV RNA detection in persons who were antibody positive (a measure commonly known as the “viremic rate” [21, 27]) in the general population, to calculate the number of HCV chronically-infected persons. The pooled mean viremic rate was obtained by performing a meta-analysis of all studies reporting measures of HCV RNA prevalence among persons who were antibody positive in the general population of Pakistan. The population size of each province was attained from the population census of the Pakistan Bureau of Statistics for 2017 [39, 40].

### HCV prevalence predictors, trends, and sources of heterogeneity

Random-effects meta-regression analyses (univariable and multivariable), following established methodology [29], were performed to determine predictors and trends of HCV prevalence, as well as sources of between-study heterogeneity. Two sets of meta-regressions were conducted, the first for only the general populations, while the second for all populations.

For the general populations, a priori relevant predictors included general population subpopulation, province, study site, sample size (< 100 or ≥ 100), year of data collection, and year of publication. For all populations, a priori relevant predictors included risk population, province, sample size (< 100 or ≥ 100), year of data collection, and year of publication. Variables qualified for inclusion in the final multivariable models provided the *p*-value was < 0.10. Variables were determined to be statistically significant in the final multivariable meta-regression provided the p-value was < 0.05.

All missing observations were imputed for the year of data collection (16.6% of all studies) by using the median of the results from subtracting the year of data collection from the year of publication. A sensitivity analysis was performed using the imputed and non-imputed observations, revealing no impact of the imputation on study results.

Meta-regressions were performed using STATA 13, through the metan command [41].

### Genotype diversity

For each province, the frequency of each HCV genotype was calculated. Persons infected with an HCV genotype that was untypeable were excluded. Persons infected with more than one HCV genotype were counted individually in the number of each genotype. Shannon Diversity Index was calculated to assess the diversity in HCV genotype distribution [42], with more diversity indicated by a higher score. The largest Shannon Diversity Index score achievable is 1.95— given the existence of seven main HCV genotypes [43].

## Results

### Search results

Additional file 1 Figure S2 describes the selection process of HCV incidence and prevalence studies in Pakistan, per the PRISMA flow diagram. Overall, 1621 citations were identified: 589 from PubMed and 1032 from Embase. After duplicates were removed and titles and abstracts were screened a total of 453 reports were identified, which subsequently underwent full-text screening. Eventually, 277 eligible reports were identified, yielding two incidence measures and 309 overall prevalence studies that included a total of 418 stratum-specific prevalence measures.

Relative to the previous systematic review [9], we identified one additional incidence study and 46 additional prevalence studies. The new incidence study was conducted in Punjab on patients initiating dialysis with a seroconversion risk of 48.9% after 18 months of follow-up [44].

All 1621 citations underwent an additional independent screening for HCV genotypes. Additional file 1: Figure S3 shows the PRISMA flow diagram. After duplicates were excluded and title and abstract screened, 74 reports underwent full-text screening. Eventually, 48 reports were included in this systematic review of HCV genotypes.

### Pooled mean HCV prevalence in the general population

**Fig. 2:**
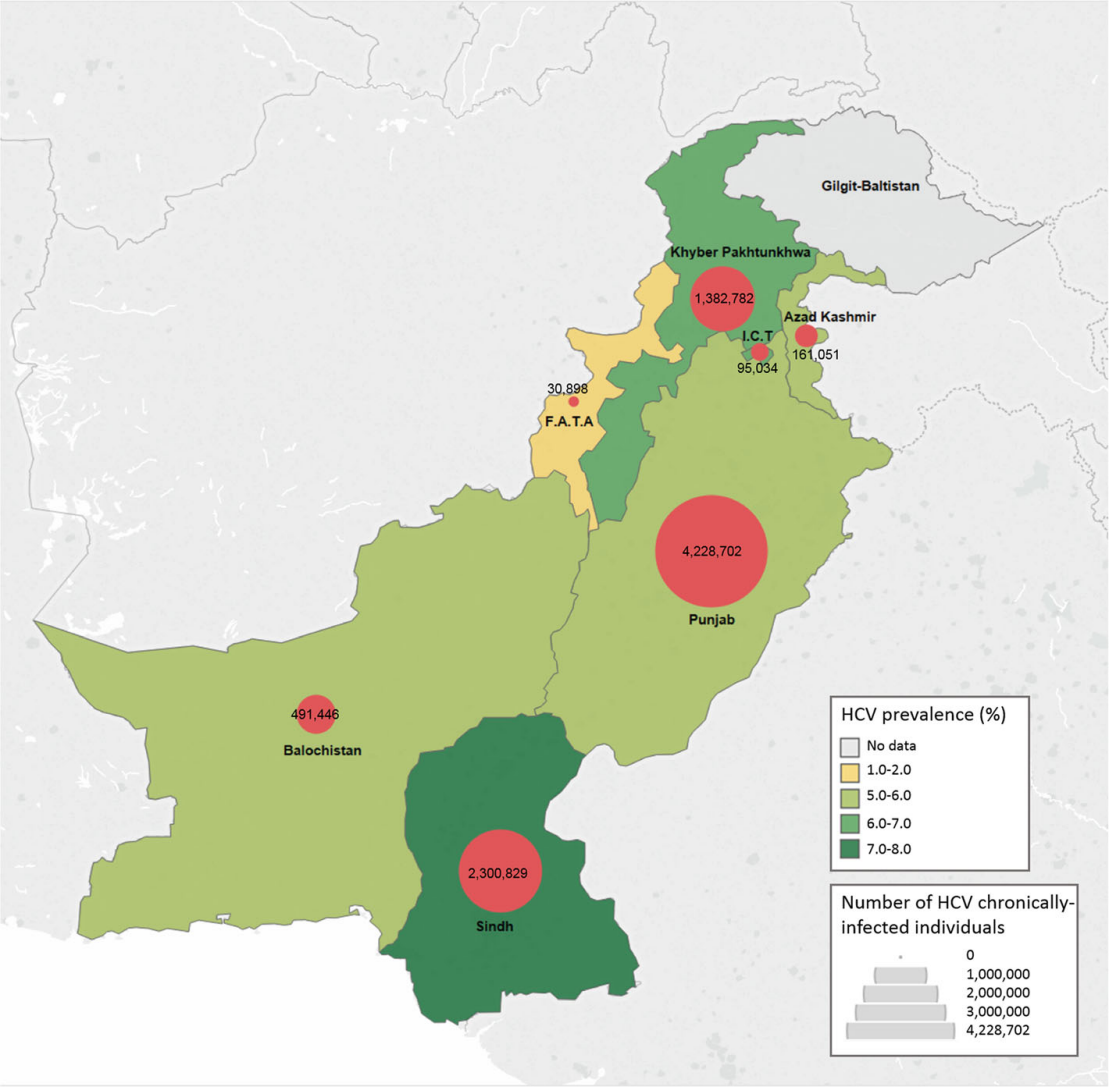
Map of the pooled mean hepatitis C virus (HCV) prevalence distribution, and the number of chronically-infected persons, across provinces of Pakistan. Footnote (Abbreviations: HCV, hepatitis C virus; F.A.T.A, Federally Administered Tribal Areas; I.C.T, Islamabad Capital Territory)

The pooled estimates for the mean HCV prevalence in the general population (Table 1, Fig. 2) was highest in Sindh at 7.0% (95% confidence interval (CI): 5.8–8.3%), followed by Islamabad Capital Territory at 6.9% (95% CI: 2.6–13.0%), Khyber Pakhtunkhwa at 6.6% (95% CI: 5.6–7.7%), Azad Kashmir at 5.8% (95% CI: 4.5–7.1%), Balochistan at 5.8% (95% CI: 2.9–9.5%), Punjab at 5.6% (95% CI: 4.5–6.8%), and F.A.T.A at 0.9% (95% CI: 0.1–2.4%). The pooled mean for Pakistan as a whole was 6.1% (96% CI: 5.5–6.7%). No studies were identified from Gilgit-Baltistan. Forest plots for the meta-analyses are in Additional file 1: Figures S4-S9.

**Table 1:** Results of the meta-analyses for hepatitis C virus (HCV) prevalence in the general population across provinces of Pakistan

Province	Studies	Samples	HCV prevalence		Pooled HCV prevalence estimate		Heterogeneity measures			Population size[36, 37]	Estimated number of HCV Ab positive persons	Estimated number of HCV chronically- infected persons
	Total N	Total n	Range (%)	Median (%)	Mean (%)	95% CI	Q (*p*-value)^a^	I^2^ (confidence limits)^b^	Prediction interval (%)^c^			
Azad Kashmir	3	9668	1.0–6.6	6.1	5.8	4.5–7.1	9.1 (*p* < 0.01)	77.9% (28.7–93.2%)	0.0–26.8	4,045,366	234,631 (182,042-287,221)	158,306 (122,823-193,788)
Balochistan	8	10,952	1.5–20.8	5.1	5.8	2.9–9.5	309.6 (*p* < 0.01)	97.7% (96.8–98.4%)	0.0–23.0	12,344,408	715,976 (357,988-1,172,719)	483,069 (241,534-791,233)
F.A.T.A	2*	11,643	0.4–1.6	–	0.9	0.1–2.4	–	–	–	5,001,676	45,015 (5002-120,040)	30,372 (3375-80,991)
Islamabad Capital Territory	5	83,642	1.7–24.6	4.8	6.9	2.6–13.0	1109.8 (*p* < 0.01)	99.6% (99.5–99.7%)	0.0–38.5	2,006,572	138,453 (52,171-260,854)	93,415 (35,200-175,998)
Khyber Pakhtunkhwa	30	255,708	0.9–73.4	6.0	6.6	5.6–7.7	1942.8 (*p* < 0.01)	98.5% (98.3–98.7%)	2.2–13.1	30,523,371	2,014,542 (1,709,309-2,350,300)	1,359,212 (1,153,271-1,585,747)
Punjab	64	718,771	0.0–23.8	5.2	5.6	4.5–6.8	25,417.1 (*p* < 0.01)	99.8% (99.7–99.8%)	0.1–18.2	110,012,442	6,160,697 (4,950,560-7,480,846)	4,156,622 (3,340,143-5,047,327)
Sindh	58	687,812	0.4–51.0	5.2	7.0	5.8–8.3	17,830.5 (*p* < 0.01)	99.7% (99.7–99.7%)	0.7–19.0	47,886,051	3,352,024 (2,777,391–3,974,542)	2,455,463 (2,067,758-2,907,785)
Pakistanǂ	182	2,099,434	0.0–73.4	5.2	6.1	5.5–6.7	49,464.9 (*p* < 0.01)	99.6% (96.6–96.6%)	0.8–15.7	211,819,886	12,921,013 (11,650,094-14,191,932)	8,717,808 (7,860,318-9,575,297)

Abbreviations: *CI* confidence interval, *HCV* hepatitis C virus, *Ab* antibody, *F.A.T.A* Federally Administered Tribal Areas

^a^Q: Cochran Q statistic assessing the existence of heterogeneity in HCV prevalence estimates

^b^I^2^: a measure assessing the magnitude of between-study variation that is due to difference in HCV prevalence estimates across studies rather than chance

^c^Prediction interval: a measure estimating the 95% interval in which the true HCV prevalence in a new study will lie

*Weighted average calculated as too few studies (< 3) to perform a meta-analysis

ǂExcluding the province of Gilgit-Baltistan, as no HCV prevalence data nor population size data was available for this province

All meta-analyses exhibited statistically significant heterogeneity in HCV prevalence measures (*p*-value for Cochrane’s Q statistic was always < 0.01; Table 1). Most variability originated from true variation in prevalence across studies rather than chance (I^2^ > 77.9%; Table 1).

### Estimated number of HCV infected persons

Based on a total of 11 studies identified through the systematic review (Additional file 1: Table S2), the pooled mean viremic rate for Pakistan was estimated at 67.5%. This estimate was used in calculating the number of chronic infections in the country. There was insufficient number of studies to estimate the pooled mean viremic rate for each province individually.

Table 1 reports the number of HCV antibody positive persons as well as the number of chronically-infected persons by province, and in Pakistan as a whole. The highest number of chronic infections was found in Punjab at approximately 4.2 million, followed by Sindh at 2.5 million, Khyber Pakhtunkhwa at 1.4 million, Balochistan at 0.5 million, Azad Kashmir at 0.2 million, Islamabad Capital Territory at 93,415, and F.A.T.A at 30,372.

### HCV prevalence predictors, trends, and sources of heterogeneity

#### General population

Results of the meta-regressions for the general population are in Table 2. Univariable analyses found all variables to be statistically-significant predictors (*p* < 0.1), except for year of data collection and year of publication—no evidence was found for a temporal variation in HCV prevalence.

**Table 2:** Univariable and multivariable meta-regression models for hepatitis C virus (HCV) prevalence in the general population in Pakistan

		Number of studies	Univariable analysis			Multivariable analysis^a^	
			OR (95% CI)	*p*-value	Variance explained adjusted R^2^ (%)	AOR (95% CI)	*p*-value
General population subpopulations	Blood donors	73	1	–		1	–
	Pregnant women/ANC attendees	21	2.0 (1.2–3.2)	0.007		2.1 (0.5–9.1)	0.324
	Non-specific general populations	65	3.3 (2.4–4.6)	< 0.001		3.2 (1.6–6.4)	< 0.001
	Children	5	0.6 (0.2–1.5)	0.266		0.6 (0.2–1.6)	0.299
	Outpatients	9	4.8 (2.4–9.7)	< 0.001		5.3 (2.1–13.0)	< 0.001
	Military personnel	6	0.7 (0.3–1.5)	0.322		0.7 (0.3–2.1)	0.572
	Refugees	3	2.6 (0.8–8.3)	0.111	26.2	1.9 (0.2–15.6)	0.549
Province	Punjab	64	1	–		1	–
	Azad Kashmir	3	0.8 (0.2–3.1)	0.735		0.7 (0.2–2.5)	0.544
	Balochistan	8	1.0 (0.4–2.5)	0.932		0.9 (0.4–2.0)	0.856
	F.A.T.A	2	0.2 (0.0–0.9)	0.041		0.3 (0.1–1.3)	0.099
	Islamabad Capital Territory	5	1.2 (0.4–3.5)	0.737		1.4 (0.5–4.1)	0.490
	Khyber Pakhtunkhwa	30	1.2 (0.7–2.1)	0.397		1.0 (0.6–1.7)	0.886
	Sindh	58	1.2 (0.8–1.8)	0.488		1.3 (0.9–1.8)	0.245
	Mixed/Unspecified	12	0.8 (0.8–1.8)	0.560	0.0	1.0 (0.6–2.0)	0.879
Study site	Blood bank	50	1	–		1	–
	Community	85	2.4 (1.6–3.5)	< 0.001		0.9 (0.5–1.9)	0.849
	Clinical setting	21	1.5 (0.9–2.7)	0.141		0.7 (0.3–1.3)	0.228
	ANC clinic	17	2.0 (1.1–3.5)	0.028		0.8 (0.2–4.0)	0.777
	Refugee camp	2	3.0 (0.6–14.4)	0.178	8.7	1.6 (0.1–19.3)	0.718
Sample size	< 100	8	1	–		1	–
	≥100	174	0.2 (0.1–0.5)	< 0.001	6.1	0.4 (0.2–0.8)	0.008
Year of data collection		182	1.0 (1.0–1.0)	0.413	0.0	–	
Year of publication		182	1.0 (1.0–1.1)	0.565	0.0	–	

Abbreviations: *OR* odds ratio, *AOR* adjusted odds ratio, *CI* confidence interval, *F.A.T.A* Federally Administered Tribal Areas, *ANC* Antenatal Care

^a^The adjusted R-squared for the full model was 27.5%

In the final multivariable model, province and study site lost significance (*p* > 0.05). As for the general-population subpopulation, only non-specific general populations and outpatients had statistically-significant different HCV prevalence from that of blood donors—the adjusted odds ratios (AORs) were 3.2 (95% CI: 1.6–6.4; *p*-value< 0.001) and 5.3 (95% CI: 2.1–13.0; *p*-value< 0.001), respectively. There was evidence for a small-study effect, studies with a sample size ≥100 participants had an AOR of 0.4 (95% CI: 0.2–0.8; *p*-value = 0.008) compared to those with a sample size of < 100. There were, however, only eight (out of 174) studies with a sample size < 100. The final multivariable model explained 27.5% of prevalence variation, mostly through the general-population subpopulation variable.

#### All populations

Results of the meta-regressions for all populations are in Table 3. Univariable analyses found all variables to be statistically-significant predictors (*p* < 0.1), except for, once again, of year of data collection and year of publication—confirming lack of evidence for a temporal variation in HCV prevalence in this much larger database including all populations, well beyond general populations. Strikingly, the ORs for year of data collection and year of publication were 1.0 (95% CI: 1.0–1.0; *p*-value = 0.785) and 1.0 (95% CI: 1.0–1.0; *p*-value = 0.779), respectively, indicating (with the narrow confidence intervals) stable prevalence over time. It was remarkable that the population risk classification alone explained 51.6% of prevalence variation.

**Table 3:** Univariable and multivariable meta-regression models for hepatitis C virus (HCV) prevalence is all populations in Pakistan

		Number of studies	Univariable analysis			Multivariable analysis^a^	
			OR (95% CI)	*p*-value	Variance explained adjusted R^2^ (%)	AOR (95% CI)	*p*-value
Population classification	General population	182	1	–		1	–
	Populations at intermediate risk	75	2.1 (1.5–2.9)	< 0.001		2.0 (1.4–2.7)	< 0.001
	High-risk clinical populations	33	8.9 (5.6–14.1)	< 0.001		7.8 (4.8–12.7)	< 0.001
	Populations with liver-related conditions	80	24.4 (17.6–34.0)	< 0.001		22.3 (15.7–31.6)	< 0.001
	Special clinical populations	29	2.0 (1.2–3.3)	0.005		1.7 (1.0–2.8)	0.038
	PWID	19	24.8 (13.7–44.9)	< 0.001	51.8	23.8 (13.0–43.6)	< 0.001
Province	Punjab	133	1	–		1	–
	Azad Kashmir	3	0.3 (0.0–2.1)	0.220		0.6 (0.1–2.6)	0.532
	Balochistan	13	0.9 (0.3–2.5)	0.853		0.6 (0.3–1.2)	0.174
	F.A.T.A	2	0.1 (0.0–0.8)	0.029		0.1 (0.0–0.8)	0.028
	Islamabad Capital Territory	22	2.6 (1.2–5.8)	0.019		0.9 (0.5–1.6)	0.688
	Khyber Pakhtunkhwa	64	0.8 (0.5–1.3)	0.363		0.6 (0.4–0.9)	0.016
	Sindh	144	1.3 (0.9–2.0)	0.173		0.9 (0.7–1.2)	0.447
	Mixed/Unspecified	37	1.4 (0.8–2.8)	0.230	2.5	0.8 (0.5–1.3)	0.425
Sample size	< 100	77	1	–		1	–
	≥100	341	0.2 (0.2–0.4)	< 0.001	8.9	0.8 (0.6–1.1)	0.176
Year of data collection		418	1.0 (1.0–1.0)	0.785	0.0	–	–
Year of publication		418	1.0 (1.0–1.0)	0.779	0.0	–	–

Abbreviations: *OR* odds ratio, *AOR* adjusted odds ratio, *CI* confidence interval, *PWID* people who inject drugs, *F.A.T.A* Federally Administered Tribal Areas

^a^The adjusted R-squared for the full model was 52.4%

In the final multivariable model, HCV prevalence, relative to the general population, was much higher for people who inject drugs (PWID) [AOR of 23.8 (95% CI: 13.0–43.6; *p*-value< 0.001)], populations with liver-related conditions [AOR of 22.3 (95% CI: 15.7–31.6; *p*-value< 0.001)], and high-risk clinical populations [AOR of 7.8 (95% CI: 4.8–12.7; *p*-value< 0.001)]. HCV prevalence was also higher for populations at intermediate risk [AOR of 2.0 (95% CI: 1.4–2.7; *p*-value< 0.001)] and for special clinical populations [AOR of 1.7 (95% CI: 1.0–2.8; *p*-value = 0.038)].

Most differences in HCV prevalence by province lost statistical significance in the final multivariable model. Relative to Punjab, there was evidence for differences in prevalence only for F.A.T.A [AOR of 0.1 (95% CI: 0.0–0.8; *p*-value = 0.028)] and Khyber Pakhtunkhwa [AOR of 0.6 (95% CI: 0.4–0.9; *p*-value = 0.016)]. The small-study effect was also no longer statistically significant. The final multivariable model explained 52.4% of prevalence variation, mainly through the population-risk-classification variable.

### HCV genotypes

A total of 48 reports with HCV genotype data were identified, yielding 95 HCV genotype studies including 37,821 HCV RNA positive persons. Of these, 423 persons were infected with an untypeable genotype, and thus were omitted from further analysis. Only 7.0% of persons were infected with multiple genotypes, with the remaining persons being infected by a single genotype. Most genotype data were from Punjab (number of reports = 34). No genotype information was available from F.A.T.A, Islamabad Capital Territory, and Gilgit-Baltistan.

**Fig. 3:**
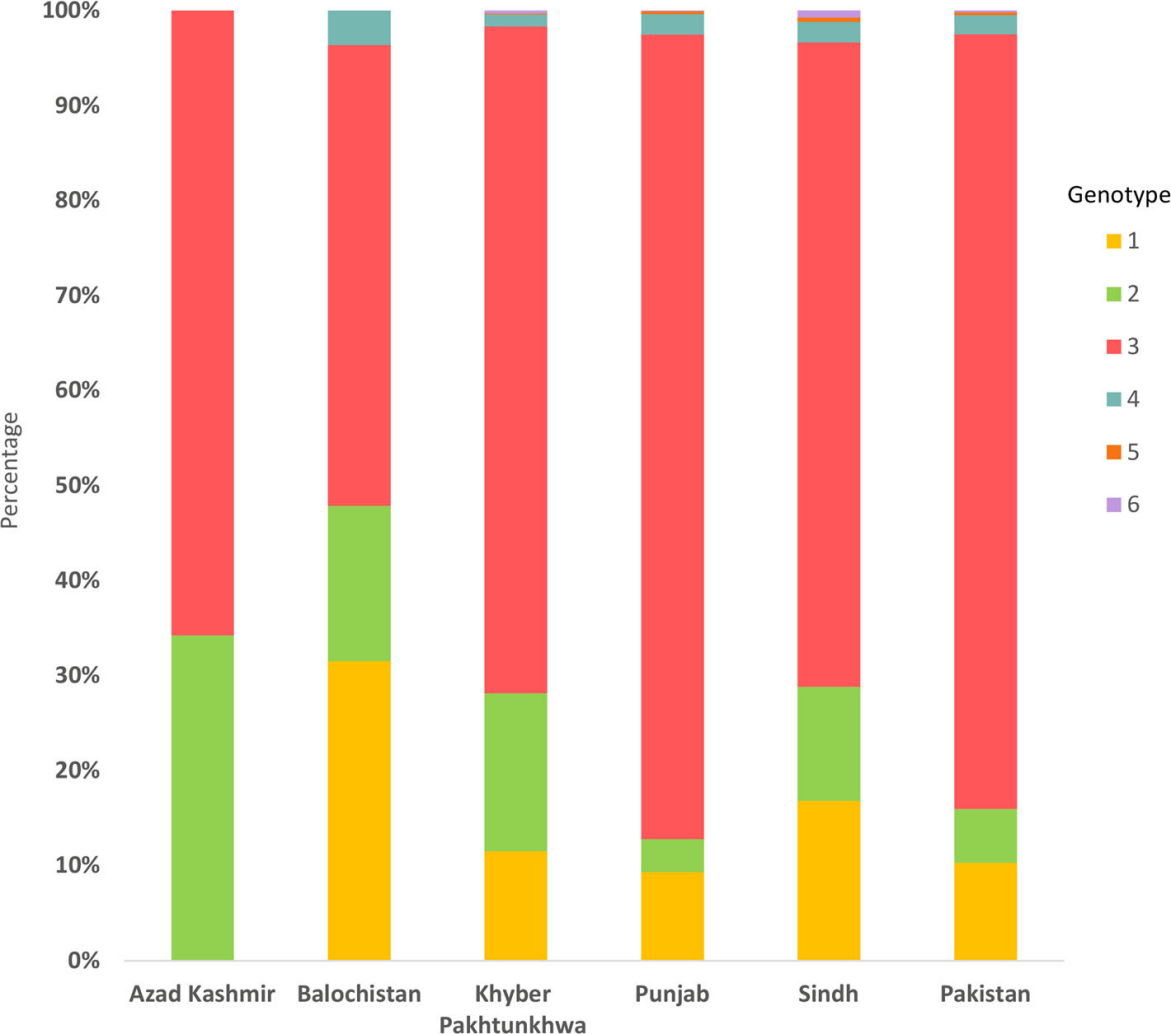
The distribution of hepatitis C virus (HCV) genotypes across provinces of Pakistan

The HCV genotype distribution and its diversity for all of Pakistan and by province, are found in Fig. 3 and Table 4, respectively, with the diversity calculated using Shannon Diversity Index (H). For all of Pakistan, the distribution demonstrated a high frequency of genotype 3 (81.5%), followed by genotype 1 (10.3%), genotype 2 (5.7%), genotype 4 (2.0%), genotype 5 (0.3%), and genotype 6 (0.2%). Genotype 7 was not identified by any study. Genotype diversity was rather low, but somewhat varied across Pakistan. The highest diversity was observed in Balochistan (H = 1.13 out of 1.95; 58.2%), followed by Sindh (H = 1.0 out of 1.95; 49.4%), Khyber Pakhtunkhwa (H = 0.88 out of 1.95; 45.0%), Azad Kashmir (H = 0.64 out of 1.95; 33.0%), and Punjab (H = 0.58 out of 1.95; 30.0%). Collectively, genotype diversity was low in Pakistan as a whole (H = 0.67 out of 1.95; 34.5%).

**Table 4:** Frequency, distribution, and Shannon Diversity Index of hepatitis C virus (HCV) genotypes across provinces of Pakistan

Country	Azad Kashmir n (%)	Balochistan n (%)	Khyber Pakhtunkhwa n (%)	Punjab n (%)	Sindh n (%)	Pakistan n (%)
Genotype 1	–	52 (31.5%)	416 (11.5%)	2674 (9.3%)	571 (16.9%)	3861 (10.3%)
Genotype 2	74 (34.3%)	27 (16.4%)	602 (16.6%)	996 (3.5%)	405 (12.0%)	2120 (5.7%)
Genotype 3	142 (65.7%)	80 (48.5%)	2538 (70.2%)	24,331 (84.7%)	2297 (67.8%)	30,484 (81.5%)
Genotype 4	–	6 (3.6%)	46 (1.3%)	615 (2.1%)	72 (2.1%)	754 (2.0%)
Genotype 5	–	–	4 (0.1%)	87 (0.3%)	16 (0.5%)	109 (0.3%)
Genotype 6	–	–	11 (0.3%)	26 (0.1%)	25 (0.7%)	70 (0.2%)
Genotype 7	–	–	–	–	–	–
Total	216 (0.6%)	165 (0.4%)	3617 (9.7%)	28,729 (76.8%)	3386 (9.1%)	37,398 (100%)
Shannon Diversity Index (H)	0.64	1.13	0.88	0.58	0.96	0.67
Index relative to total possible diversity	33.0%	58.2%	45.0%	30.0%	49.4%	34.5%

## Discussion

Based on a systematically-assembled and large database of HCV data from Pakistan, we provided in-depth quantitative assessments of diverse aspects of the HCV epidemic in this country, the second largest worldwide [10, 11, 45]. We estimated that there are 12.9 million persons who have been infected with HCV in Pakistan, 8.7 million of whom are chronically-infected—an estimate higher but in broad agreement with existing estimates ranging between 6.7–7.8 million chronic infections [10, 46, 47].

Notably and surprisingly, there was no evidence for any change in HCV prevalence over time (Tables 2, 3), contrary to other countries where several factors, such as implementation of blood supply screening and injection safety, have led to a general global trend of declining HCV prevalence [11, 25, 48]. Our results suggested that HCV prevalence in Pakistan has been stable over the past three decades, or if not, just slightly declining or slightly increasing. This finding corroborates recent modeling studies that suggested slightly decreasing [10] or slightly increasing HCV prevalence [46], and highlights the gravity of this large epidemic with substantial ongoing HCV transmission to this day.

Remarkably, the results further demonstrated minor, non-consequential, differences in HCV prevalence across Pakistan’s provinces (Tables 1, 2, 3 and Fig. 2). Collectively, the results indicate a largely pervasive and homogenous epidemic that impacted all parts of the country, including the Punjab and Sindh provinces that contribute most of Pakistan’s population [39]. Indeed, Punjab and Sindh combined were estimated to host approximately 75% of all chronic infections in this country (Table 1).

The identified spatial homogeneity of the epidemic appears to contradict some of the results of Pakistan’s single national survey that was conducted in 2007–2008 [12]. The survey identified considerable variations in prevalence across provinces, ranging from 1.1% in Khyber Pakhtunkhwa to 6.7% in Punjab [12]. Meanwhile, our results suggest only small variations in prevalence (Table 1). Furthermore, our estimate for the pooled mean HCV prevalence for Pakistan as a whole, at 6.1% (Table 1), is higher than the result of the national survey at 4.8% [12].

Genotype 3 was found to be the most common genotype in all of Pakistan’s provinces (Fig. 3 and Table 4), supporting further the pervasive and homogenous nature of the epidemic. This finding also corroborates overlapping and linked HCV transmission networks across Pakistan’s provinces. Only minor variations in genotype distribution were found across provinces (Fig. 3 and Table 4), and these appeared to reflect transmission links with neighboring countries [11, 23]. Balochistan had the highest frequency of genotype 1 (31.5%), but borders Iran, a country in which genotype 1 is the dominant circulating genotype [23]. Similarly, Punjab and Khyber Pakhtunkhwa had the highest frequencies of genotype 3 (84.7 and 70.2%, respectively), but have shared borders with Afghanistan to the West and India to the East, countries in which genotype 3 is the dominant circulating strain [11, 23]. Of note that genotype 2 was present at > 10% in all provinces, excluding Punjab, in a context of some evidence suggesting increasing frequency of this genotype in Pakistan [49, 50].

A key finding of this study is that population risk classification alone explained over 50% of prevalence variation (Table 3). HCV prevalence was much higher in PWID, people with liver-related conditions, and high-risk clinical populations, than in the general population (Table 3). This finding is remarkable for a country that has a high-prevalence generalized HCV epidemic. The finding demonstrates how HCV infection is associated with identifiable risk factors that map the contours of the epidemiology, and points to a need for targeted interventions, even in countries with generalized epidemics. The finding also affirms the role of injecting drug use and healthcare exposures in transmission networks, and testifies to the prominent role that this infection is playing in liver disease burden in this country. Furthermore, this finding provides avenues to optimize screening in the immediate future, targeting it to subpopulations with higher prevalence, alleviating one of the major obstacles to current HCV elimination programs in Pakistan—identifying infected individuals in a country with a generalized HCV epidemic [28, 45, 51].

A marked scale-up in patients treated with DAAs in Pakistan was observed from 2015 (approximately 65,000 patients) to 2016 (approximately 161,000 patients) [52], due in part to drastically reduced prices for DAAs [45] (currently available at US$35 per full treatment course [53]). In spite of this, the proportion of patients treated was still very low at only 1.8% in 2016, emphasizing the necessity to plan and implement a mass-scale increase in diagnosis and treatment to meet WHO targets [45]. To address the high burden of HCV and achieve the WHO target to eliminate this infection by 2030, Pakistan’s National Hepatitis Strategic Framework (NHSF) 2017–2021 was developed [54]. The NHSF, informed by key national and international partners and stakeholders, including WHO, outlines testing and treatment operational targets to achieve WHO elimination goal, and stresses optimization of access to DAA treatments at an affordable cost [54]. To this end, on a provincial level, ambitious initiatives have been launched, such as The Punjab Hepatitis Act 2018 [55], which could play a significant role in controlling hepatitis at a provincial level.

As for limitations, no HCV prevalence data were identified for the small province of Gilgit-Baltistan. There was also variability in availability of HCV prevalence data and genotype data by province—most genotype data were from Punjab (76.8%; Table 4). Some of the provinces had only a small number of studies, and most studies for all provinces used convenience sampling, which may have affected the representativeness of some of the estimates. Only PubMed and Embase, the two canonical international databases, were searched, but other data, such as country-level data of routine testing or grey literature, may exist but were not factored in generating our estimates.

A key gap in Pakistan’s data was identified. Despite the sizable and spatially pervasive nature of this epidemic, only one national probability-based survey was conducted, and over a decade ago [12]. Repeating this survey is critical in improving the estimates for prevalence and number of infections, assessing HCV spatial distribution and temporal trends, and highlighting key risk factors and drivers of incidence, as has been done in other countries [56–64].

## Conclusion

Pakistan’s HCV epidemic shows homogeneity across the provinces, and over time. HCV prevalence is persistent at a high level, with no evidence for a decline over the last three decades. Genotype 3 is the most common genotype in all of Pakistan’s provinces, with only minor differences in genotype distribution by province. Very high HCV prevalence levels were identified in PWID, populations with liver-related conditions, and high-risk clinical populations. A new national survey for HCV infection is critical to elucidate and update our understanding of the epidemic, and to inform the development of targeted, cost-effective interventions in Pakistan. Scale up of HCV treatment and prevention is urgently needed to eliminate HCV infection by 2030, per the WHO global target.

## Additional file

Additional file 1: ‘Characterization of the hepatitis C virus in Pakistan’. (DOCX 1618 kb)

## Data Availability

All data used and analyzed during this study is based on a systematic review of existing literature and is available from the author, Dr. Laith J. Abu-Raddad, by request.

## Abbreviations

AOR: Adjusted odds ratio
CI: Confidence interval
DAA: Direct-acting antivirals
F.A.T.A: Federally Administered Tribal Areas
HCV: Hepatitis C virus
MENA: Middle East and North Africa
PRISMA: Preferred Reporting Items for Systematic Reviews and Meta-analyses
PWID: People who inject drugs
RNA: Ribonucleic acid
WHO: World Health Organization
