# Supplementary material for: Characterization of the hepatitis C virus epidemic in Pakistan
Source: BMC Infect Dis. 2019 Sep 14;19:809. doi: 10.1186/s12879-019-4403-7 (PMC6744714; doi:10.1186/s12879-019-4403-7)
Supplement: Supplementary file 1 — ‘Characterization of the hepatitis C virus in Pakistan’. (DOCX 1618 kb) [file 12879_2019_4403_MOESM1_ESM.docx]

**SUPPLEMENTARY MATERIAL**

**Characterization of the hepatitis C virus epidemic in Pakistan**

Sarwat Mahmud^1^, Zaina Al Kanaani^1^, and Laith J. Abu-Raddad^1, 2, 3*^

^1^*Infectious Disease Epidemiology Group, Weill Cornell Medicine-Qatar, Cornell University, Qatar Foundation - Education City, Doha, Qatar*

^2^*Department of Healthcare Policy & Research, Weill Cornell Medicine, Cornell University, New York, USA*

^3^College of Health and Life Sciences, Hamad bin Khalifa University, Doha, Qatar

**Table S1.** Preferred Reporting Items for Systematic Reviews and Meta-analyses (PRISMA) checklist [1].

| **Section/topic** | **#** | **Checklist item** | **Reported in main text on** |
| --- | --- | --- | --- |
| **TITLE** | | |  |
| Title | 1 | Identify the report as a systematic review, meta-analysis, or both. | p. 1 |
| **ABSTRACT** | | |  |
| Structured summary | 2 | Provide a structured summary including, as applicable: background; objectives; data sources; study eligibility criteria, participants, and interventions; study appraisal and synthesis methods; results; limitations; conclusions and implications of key findings; systematic review registration number. | p. 2 |
| **INTRODUCTION** | | |  |
| Rationale | 3 | Describe the rationale for the review in the context of what is already known. | p. 4 |
| Objectives | 4 | Provide an explicit statement of questions being addressed with reference to participants, interventions, comparisons, outcomes, and study design (PICOS). | p.4-5 |
| **METHODS** | | |  |
| Protocol and registration | 5 | Indicate if a review protocol exists, if and where it can be accessed (e.g., Web address), and, if available, provide registration information including registration number. | p. 5 |
| Eligibility criteria | 6 | Specify study characteristics (e.g., PICOS, length of follow-up) and report characteristics (e.g., years considered, language, publication status) used as criteria for eligibility, giving rationale. | [2] |
| Information sources | 7 | Describe all information sources (e.g., databases with dates of coverage, contact with study authors to identify additional studies) in the search and date last searched. | p. 5 |
| Search | 8 | Present full electronic search strategy for at least one database, including any limits used, such that it could be repeated. | Figure S1 |
| Study selection | 9 | State the process for selecting studies (i.e., screening, eligibility, included in systematic review, and, if applicable, included in the meta-analysis). | p. 4, Figure S2 |
| Data collection process | 10 | Describe method of data extraction from reports (e.g., piloted forms, independently, in duplicate) and any processes for obtaining and confirming data from investigators. | p. 5, [2] |
| Data items | 11 | List and define all variables for which data were sought (e.g., PICOS, funding sources) and any assumptions and simplifications made. | p. 4-5, [2] |
| Risk of bias in individual studies | 12 | Describe methods used for assessing risk of bias of individual studies (including specification of whether this was done at the study or outcome level), and how this information is to be used in any data synthesis. | [2] |
| Summary measures | 13 | State the principal summary measures (e.g., risk ratio, difference in means). | p. 5 |
| Synthesis of results | 14 | Describe the methods of handling data and combining results of studies, if done, including measures of consistency (e.g., I^2^) for each meta-analysis. | p. 6-7 |
| Risk of bias across studies | 15 | Specify any assessment of risk of bias that may affect the cumulative evidence (e.g., publication bias, selective reporting within studies). | p. 6-7 |
| Additional analyses | 16 | Describe methods of additional analyses (e.g., sensitivity or subgroup analyses, meta-regression), if done, indicating which were pre-specified. | p.6-8 |
| **RESULTS** | | |  |
| Study selection | 17 | Give numbers of studies screened, assessed for eligibility, and included in the review, with reasons for exclusions at each stage, ideally with a flow diagram. | p.8-9, Figure S2 |
| Study characteristics | 18 | For each study, present characteristics for which data were extracted (e.g., study size, PICOS, follow-up period) and provide the citations. | [2] |
| Risk of bias within studies | 19 | Present data on risk of bias of each study and, if available, any outcome level assessment (see item 12). | [2] |
| Results of individual studies | 20 | For all outcomes considered (benefits or harms), present, for each study: (a) simple summary data for each intervention group (b) effect estimates and confidence intervals, ideally with a forest plot. | [2] |
| Synthesis of results | 21 | Present results of each meta-analysis done, including confidence intervals and measures of consistency. | p. 9-10, Table 1 |
| Risk of bias across studies | 22 | Present results of any assessment of risk of bias across studies (see Item 15). | p. 8-10, Table 2 & 3 |
| Additional analysis | 23 | Give results of additional analyses, if done (e.g., sensitivity or subgroup analyses, meta-regression [see Item 16]). | p. 10-12, Table 2, 3 & 4 |
| **DISCUSSION** | | |  |
| Summary of evidence | 24 | Summarize the main findings including the strength of evidence for each main outcome; consider their relevance to key groups (e.g., healthcare providers, users, and policy makers). | p. 13-15 |
| Limitations | 25 | Discuss limitations at study and outcome level (e.g., risk of bias), and at review-level (e.g., incomplete retrieval of identified research, reporting bias). | p. 15 |
| Conclusions | 26 | Provide a general interpretation of the results in the context of other evidence, and implications for future research. | p. 15-16 |
| **FUNDING** | | |  |
| Funding | 27 | Describe sources of funding for the systematic review and other support (e.g., supply of data); role of funders for the systematic review. | p. 17 |

p, page(s).

**Figure S1.** Search criteria for systematically reviewing hepatitis C virus (HCV) prevalence data in Pakistan.

**PubMed (last searched: March 19, 2018)**

("Hepatitis C"[Mesh] OR "Hepatitis C Antibodies"[Mesh] OR "Hepatitis C Antigens"[Mesh] OR "Hepacivirus"[Mesh] OR "Hepatitis C"[Text] OR "Hepacivirus"[Text] OR "HCV"[Text]) AND ("Pakistan"[Mesh] or Pakistan*[text])

**Embase (last searched: March 19, 2018)**

(exp. Pakistan/ OR Pakistan*.mp.) AND (exp Hepatitis C/ OR hepatitis C.mp. OR exp hepatitis C antibody/ OR exp hepatitis C antigen/ OR exp Hepatitis C virus/ OR HCV.mp. OR hepacivirus .mp.)

**Figure S2.** Flow chart adapted from the PRISMA 2009 guidelines [1] outlining article selection process for the systematic review of hepatitis C virus (HCV) incidence and/or prevalence in Pakistan.


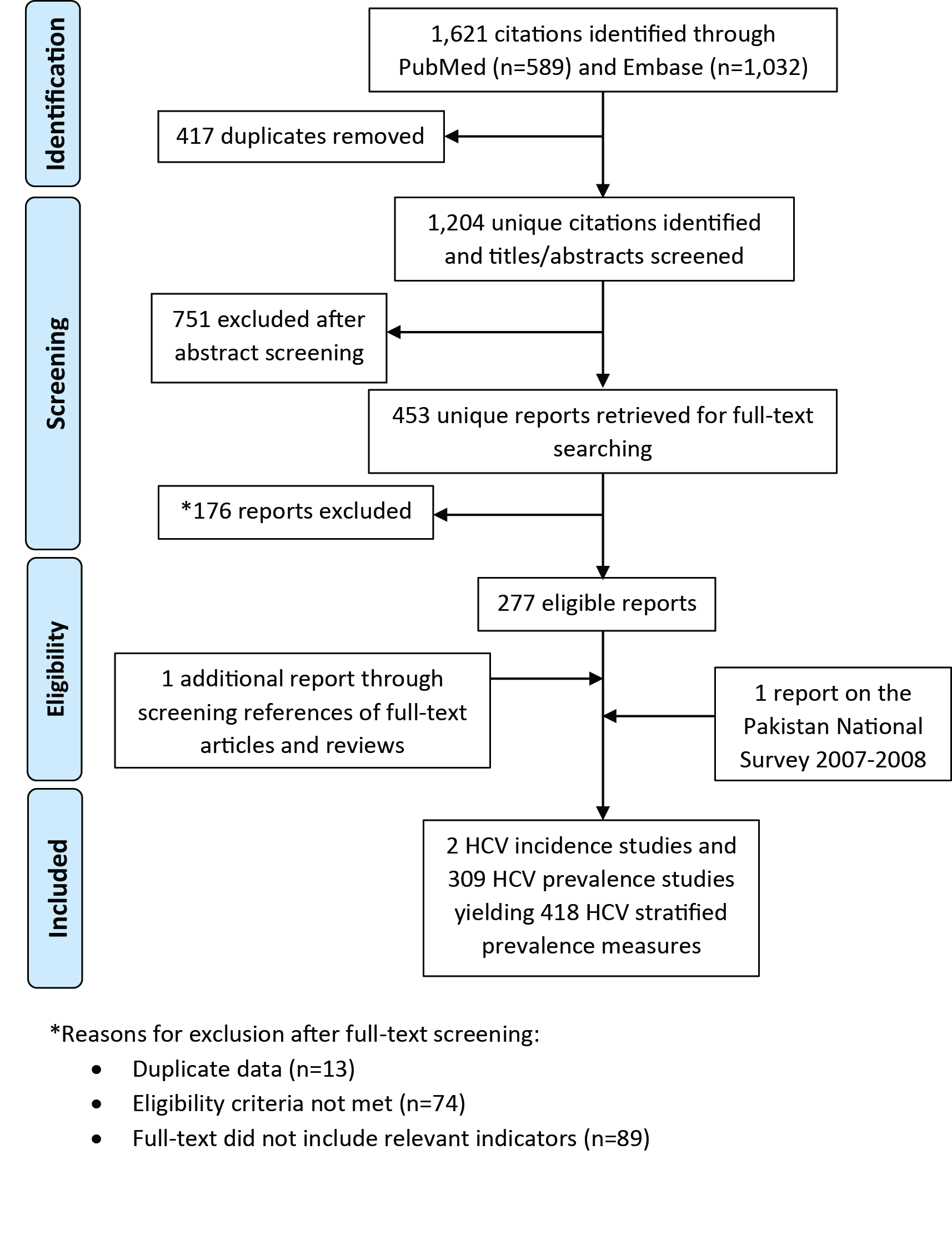


**Figure S3.** Flow chart adapted from the PRISMA 2009 guidelines [1] outlining article selection process for the systematic review of hepatitis C virus (HCV) genotypes.


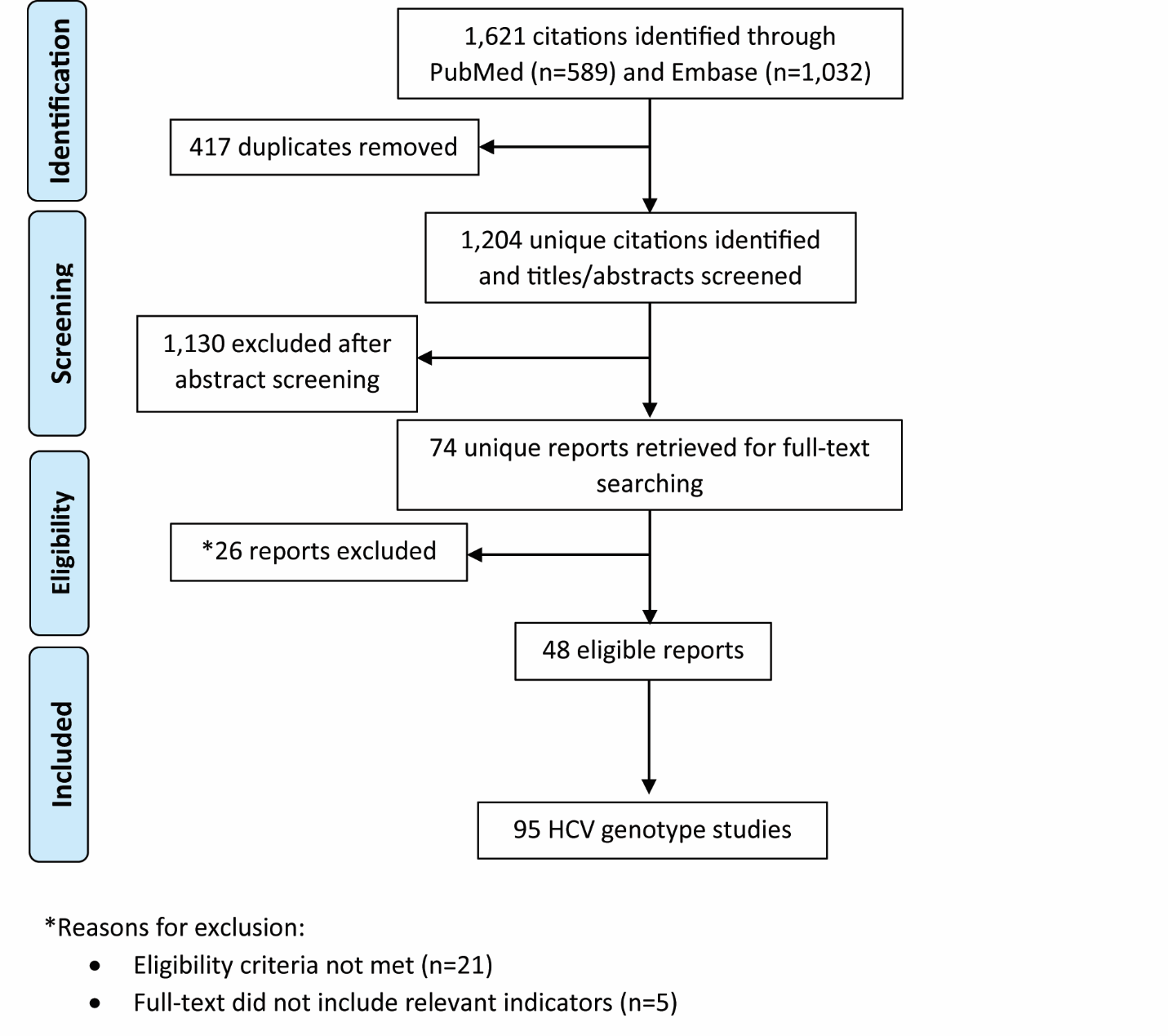


**Figure S4.** Forest plot presenting results of the meta-analysis of hepatitis C virus (HCV) prevalence in Azad Kashmir.  **
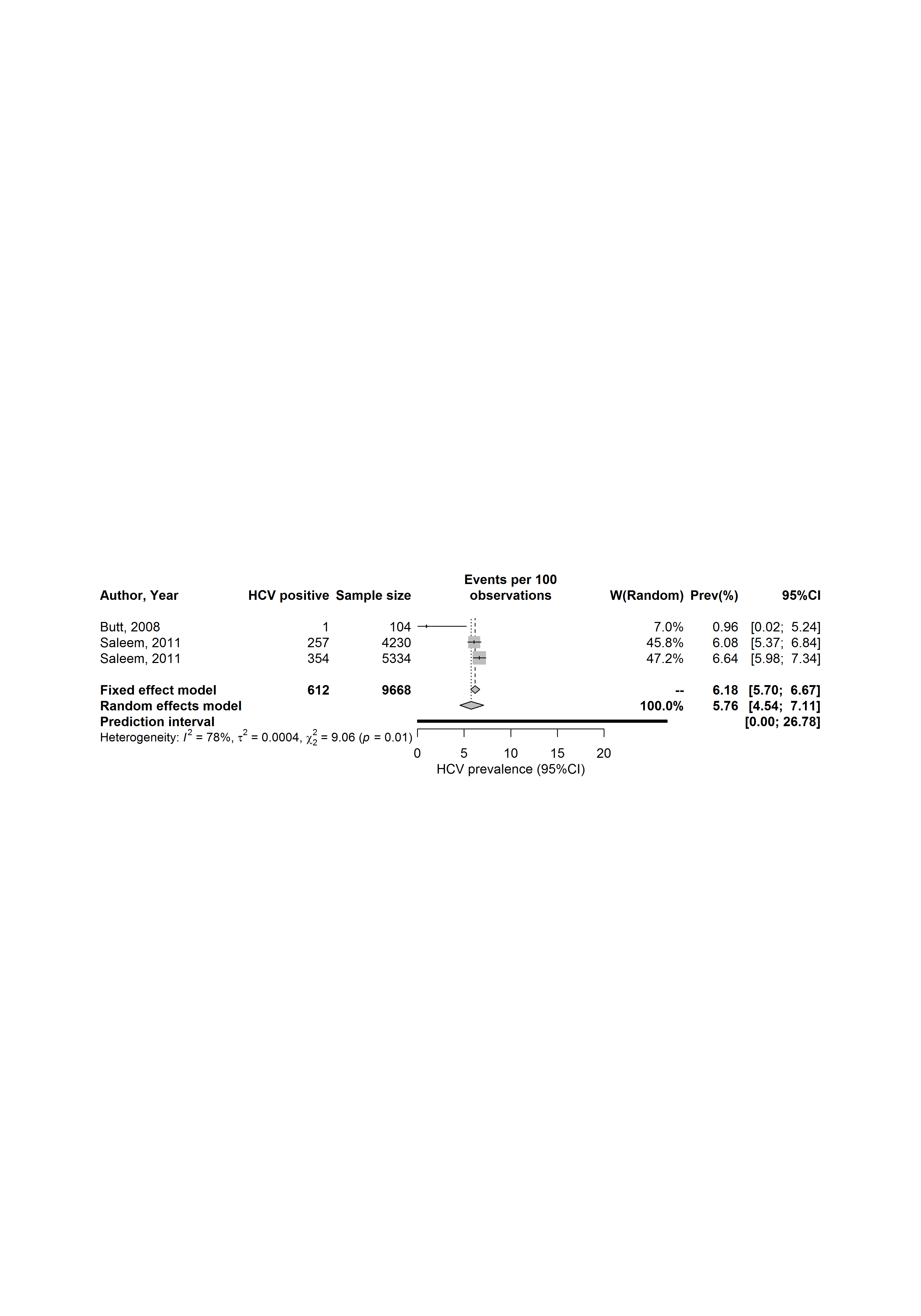
**

**Figure S5.** Forest plot presenting results of the meta-analysis of hepatitis C virus (HCV) prevalence in Balochistan.
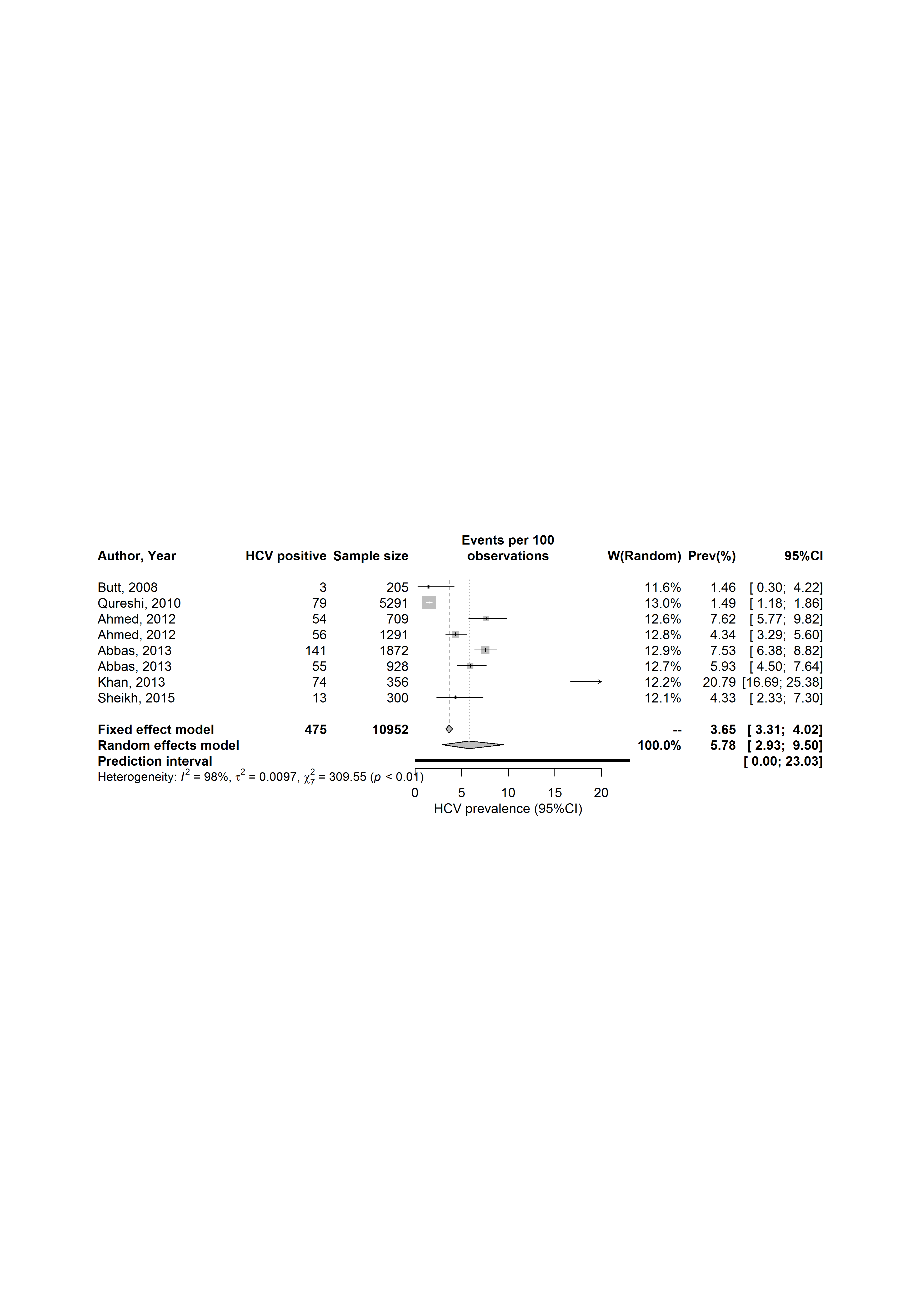


**Figure S6.** Forest plot presenting results of the meta-analysis of hepatitis C virus (HCV) prevalence in Islamabad Capital Territory.


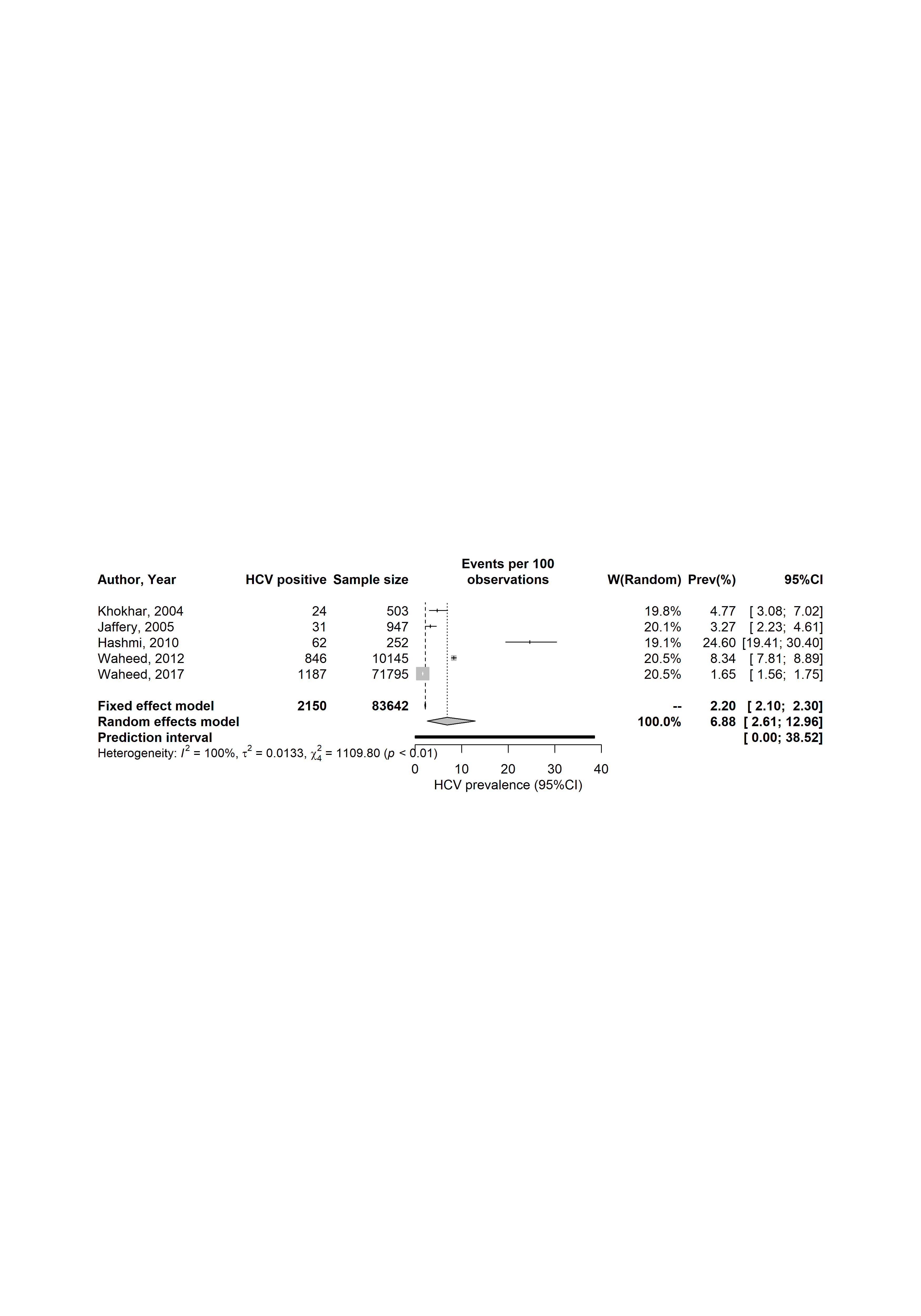


**Figure S7.** Forest plot presenting results of the meta-analysis of hepatitis C virus (HCV) prevalence in Khyber Pakhtunkhwa.
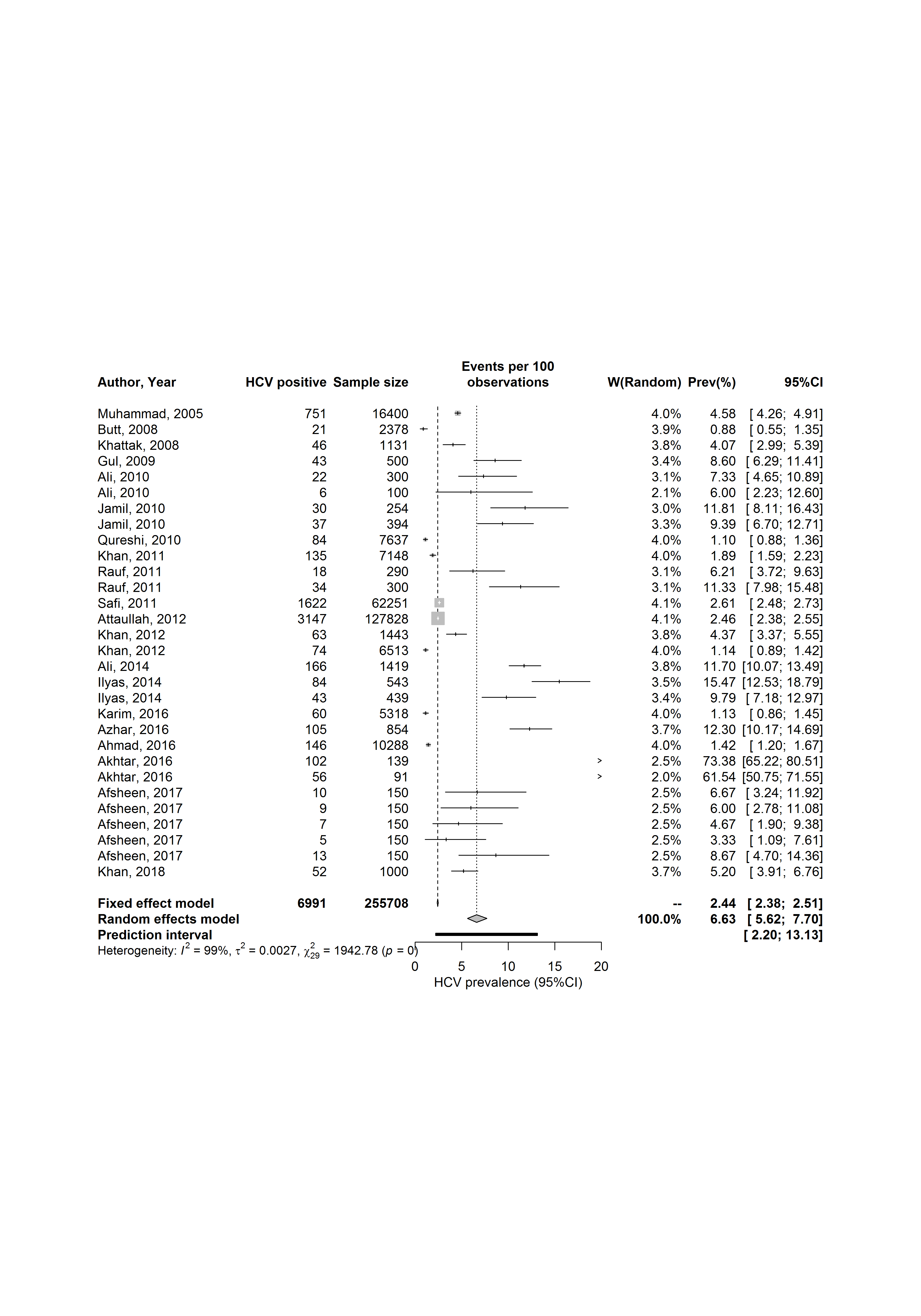


**Figure S8.** Forest plot presenting results of the meta-analysis of hepatitis C virus (HCV) prevalence in Punjab.
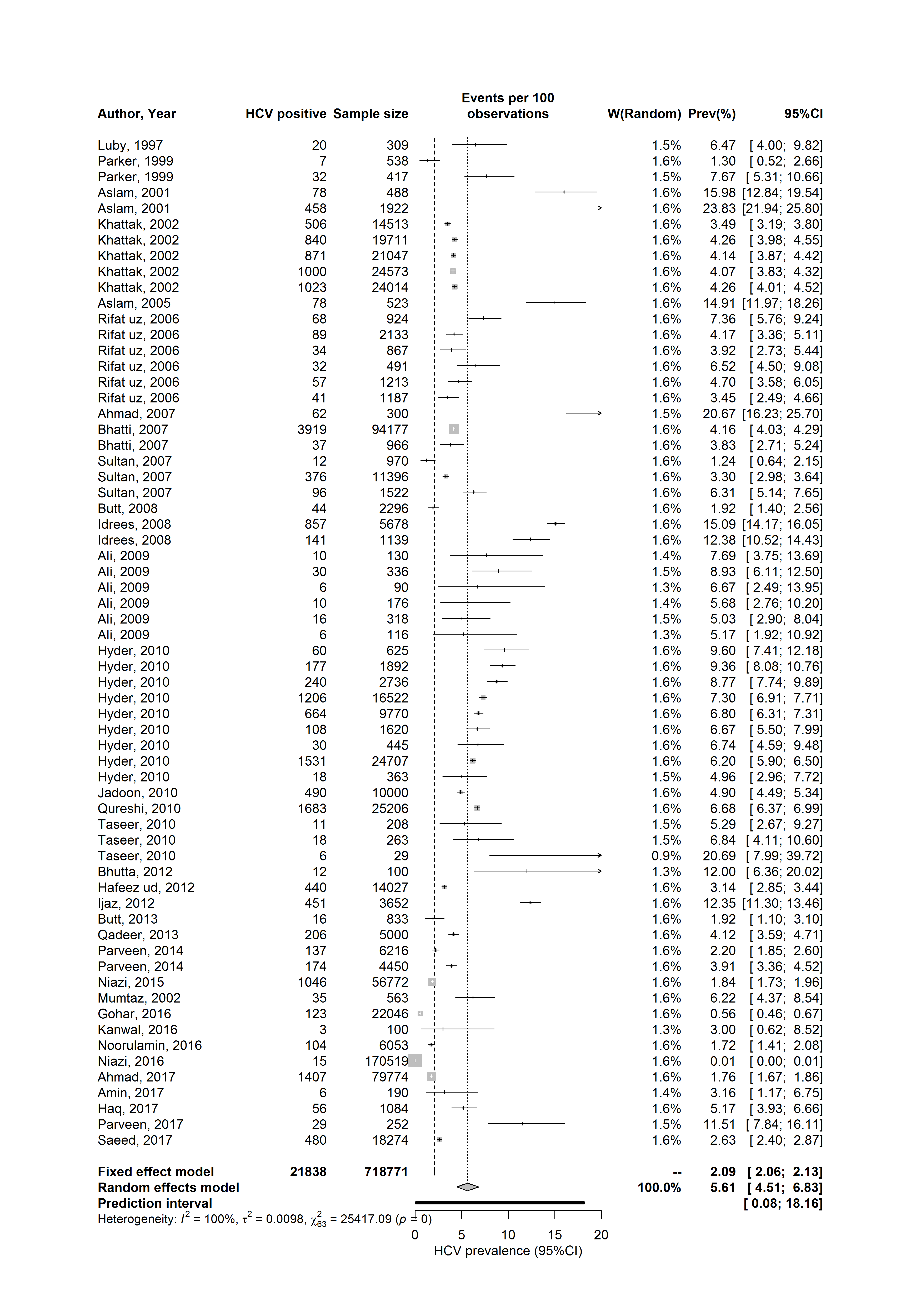


**Figure S9.** Forest plot presenting results of the meta-analysis of hepatitis C virus (HCV) prevalence in Sindh.
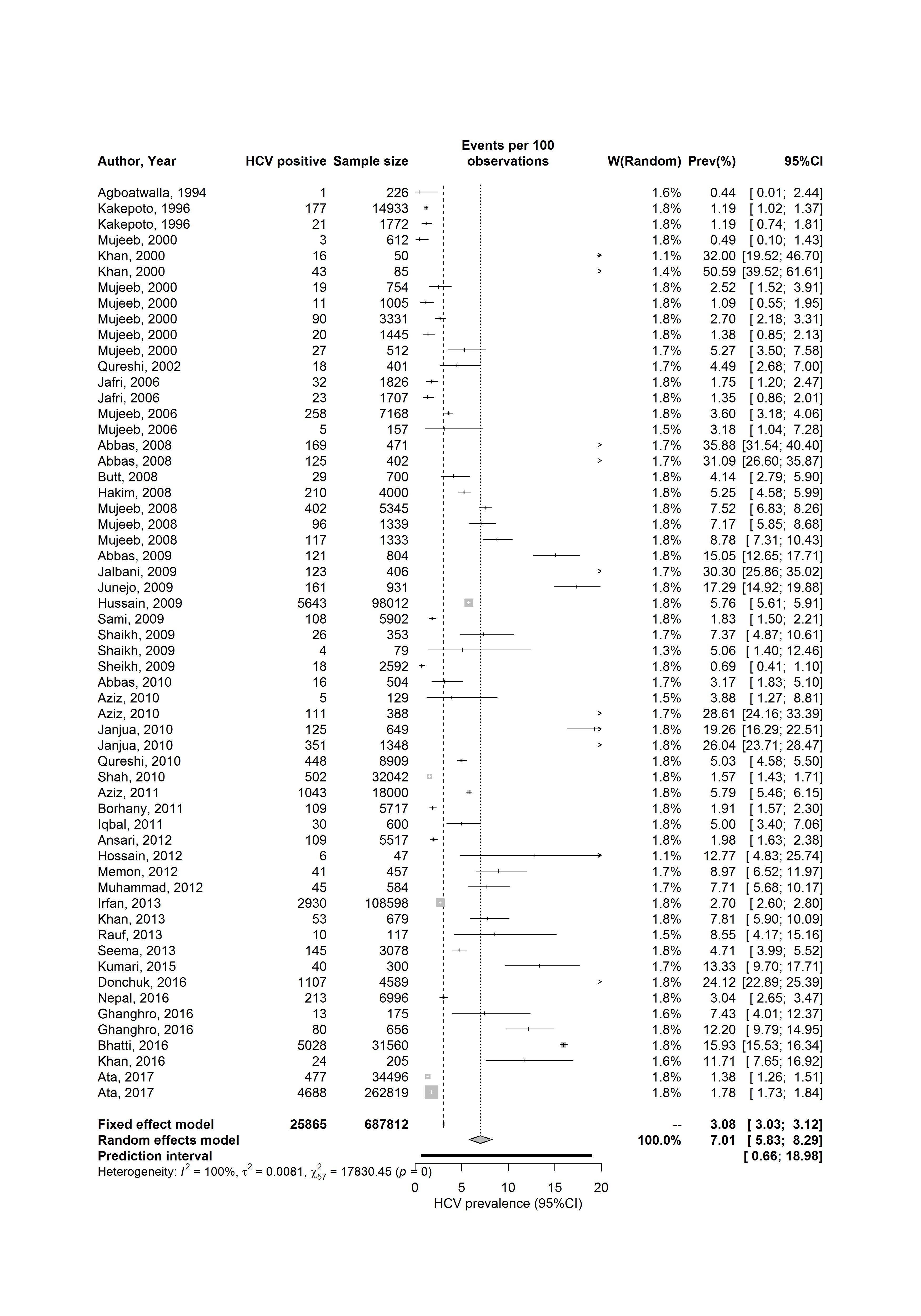


**Table S2.** Studies reporting hepatitis C virus (HCV) viremic rate in Pakistan.

| **Author, year (citation)** | **Year(s) of data collection** | **Population** | **Number of HCV antibody-positive tested for RNA** | **HCV viremic rate (%)** |
| --- | --- | --- | --- | --- |
| Aziz, 2011 [3] | 2005-2009 | Pregnant woman | 640 | 79.7 |
| Donchuk, 2016 [4] | 2015-2016 | Outpatients | 1107 | 89.0 |
| Idrees, 2008 [5] | 1999-2007 | General population | 857 | 49.2 |
| Idrees, 2008 [5] | 1999-2007 | General population | 141 | 50.4 |
| Karim, 2016 [6] | 2015 | Blood donors | 60 | 93.0 |
| Khokhar, 2004 [7] | 2001-2002 | Pregnant woman | 18 | 72.0 |
| Rauf, 2011 [8] | 2009 | Refugees | 34 | 50.0 |
| Rauf, 2011 [8] | 2012-2013 | Refugees | 18 | 44.4 |
| Bhatti, 2016 [9] | 2010-2015 | General population | 5028 | 85.8 |
| Afsheen, 2017 [10] | NS | Pregnant women | 44 | 22.7 |
| Khan, 2018 [11] | 2014-2015 | Refugees | 52 | 75.0 |

Abbreviations: RNA, ribonucleic acid.

**References**

1. Moher D, Liberati A, Tetzlaff J, Altman DG, The PG. Preferred Reporting Items for Systematic Reviews and Meta-Analyses: The PRISMA Statement. PLoS Med. 2009;6.

2. Al Kanaani Z, Mahmud S, Kouyoumjian SP, Abu-Raddad LJ. The epidemiology of hepatitis C virus in Pakistan: systematic review and meta-analyses. Royal Society open science. 2018;5(4):180257.

3. Aziz S, Hossain N, Karim SA, Rajper J, Soomro N, Noorulain W, et al. Vertical transmission of hepatitis C virus in low to middle socio-economic pregnant population of Karachi. Hepatology international. 2011;5(2):677-80.

4. Donchuk D, Rossi G, Bjorklund Y, Zainal H, Auat R. Hepatitis C treatment in a primary care clinic in the high HCV burden setting in Karachi, Pakistan. Hepatol Int. 2016;10:S34.

5. Idrees M, Lal A, Naseem M, Khalid M. High prevalence of hepatitis C virus infection in the largest province of Pakistan. Journal of digestive diseases. 2008;9(2):95-103.

6. Karim F, Nasar A, Alam I, Alam I, Hassam S, Gul R, et al. Incidence of active HCV infection amongst blood donors of Mardan District, Pakistan. Asian Pac J Cancer Prev. 2016;17(1):235-8.

7. Khokhar N, Aijazi I, Gill ML. Spectrum of hepatocellular carcinoma at Shifa International Hospital, Islamabad. Journal of Ayub Medical College Abbottabad. 2003;15(4).

8. Rauf A, Nadeem MS, Ali A, Iqbal M, Mustafa M, Muzammal Latif M, et al. Prevalence of hepatitis B and C in internally displaced persons of war against terrorism in Swat, Pakistan. The European Journal of Public Health. 2010;21(5):638-42.

9. Bhatti S, Manzoor S. Molecular epidemiology and clinical features of hepatitis c virus (HCV) in epidemic areas of interior Sindh, Pakistan. Pakistan Journal of Medical Sciences. 2016;32(5):1279-82. PubMed PMID: 620328797.

10. Afsheen Z, Ahmad B, Bashir S. Hospital-visiting pregnant women signal an increased spread of hepatitis C infection in Khyber Pakhtunkhwa region of Pakistan. Virology Journal. 2017;14 (1) (no pagination)(195). PubMed PMID: 618674608.

11. Khan A, Qazi J. Risk factors and molecular epidemiology of HBV and HCV in internally displaced persons (IDPs) of North Waziristan Agency, Pakistan. Journal of the Pakistan Medical Association. 2018;68(2):165-9. PubMed PMID: 620356378.
